# Efficacy of S-Flurbiprofen Plaster for Analgesia Following Total Hip Arthroplasty

**DOI:** 10.7759/cureus.66805

**Published:** 2024-08-13

**Authors:** Yoshiaki Miyake, Shigeru Mitani, Yoshifumi Namba, Norifumi Umehara, Toyohiro Kawamoto, Shuro Furuichi

**Affiliations:** 1 Bone and Joint Surgery, Kawasaki Medical School, Kurashiki, JPN

**Keywords:** s-flurbiprofen, postoperative analgesia, osteoarthritis, numerical rating pain intensity scale, non-steroidal anti-inflammatory drug

## Abstract

Purpose: S-flurbiprofen (SFP) plaster, a non-steroidal anti-inflammatory drug preparation that penetrates effectively into deep tissue, is currently used as a conservative treatment for osteoarthritis. We investigated the analgesic and adverse effects of SFP plaster after total hip arthroplasty (THA).

Methods: A retrospective comparative study identified 100 patients who underwent primary THA in our department. Group A consisted of 50 patients who received the selective cyclooxygenase-2 inhibitor celecoxib for 14 days after surgery, while Group B consisted of 50 patients who received SFP plaster for 14 days after surgery. We noted the numerical rating pain intensity scale (NRS) score, body temperature, and adverse effects of the analgesics.

Results: Groups A and B showed no significant difference in NRS scores (p > 0.05). The body temperature was significantly higher in Group B than in Group A on days one, two, three, and five (p < 0.01). In Group A, two patients (4%) showed drug-induced renal dysfunction, and one patient (2%) showed gastrointestinal disturbance. Patients in Group B showed no systemic or local adverse effects.

Conclusions: The application of SFP plaster after THA provided an analgesic effect similar to that obtained with oral celecoxib without causing obvious side effects. Applying an SFP plaster may be an effective solution for postoperative analgesia.

## Introduction

Total hip arthroplasty (THA) is a highly effective procedure for relieving pain and restoring function in patients with hip disease. Cases of THA have been increasing number every year worldwide [1]. The increasing popularity of muscle-sparing minimally invasive surgery (MIS) [2] has enabled patients to leave the hospital and return to normal life earlier after surgery [3], during which time postoperative pain control is important. The use of oral nonsteroidal anti-inflammatory drugs (NSAIDs) is highly prevalent in Japan. However, as NSAIDs express their anti-inflammatory effects by suppressing the cyclooxygenase (COX) system and decreasing prostaglandin production [4], they may induce adverse effects such as gastrointestinal disturbances [5,6] and renal dysfunction [7].

As conventional topical NSAIDs do not readily enter plasma [8], they show minimal systemic side effects such as gastrointestinal disturbances or renal dysfunction [9,10] and have been recommended as a conservative treatment option for osteoarthritis [11,12]. However, these topical analgesics are rarely used for postoperative analgesia since they are considered to have a low analgesic effect against acute or severe pain due to their low systemic permeability [13]. To the best of our knowledge, no previous report has evaluated the effect of topical NSAIDs on post-THA pain.

S-flurbiprofen (SFP) plaster (40 mg/140 cm^2^, LOQOAⓇ tape; Taisho Pharmaceutical Co., Tokyo, Japan) is a topical NSAID that effectively penetrates deep tissues and plasma and has been commercially available in 2016. SFP contains the active optical enantiomer (S isomer) of FP, a powerful COX inhibitor that exhibits good skin penetration. Sugimoto et al. calculated the transdermal absorption rate by determining the amount of drug remaining in tapes detached after application to rat skin for 24 hours and found that the rate for SFP was 92.9%, much greater than the rates for ketoprofen (67.8%) and loxoprofen (32.4%) [14], which are widely used as topical NSAIDs. In a study of 19 patients scheduled to undergo total knee arthroplasty (TKA), Yataba et al. applied 40 mg of SFP plaster to 10 knees and 40 mg of FP plaster to nine knees for 12 hours and compared the synovial membrane tissue, synovial fluid, and plasma samples collected during surgery. They found that, in comparison with the corresponding drug concentrations for FP plaster, the drug concentration after using SFP plaster was 14.8-fold greater in synovial membrane tissue, 32.7-fold greater in the synovial fluid, and 34.5-fold greater in plasma, indicating that SFP penetrates tissues significantly more effectively [15]. In clinical practice, a randomized blinded trial on 633 patients with osteoarthritis of the knee also showed that, when SFP plaster and FP plaster were each applied for two weeks, SFP plaster provided significantly better pain relief [16]. Moreover, a study examined the analgesic effect of SFP plaster after TKA [17]; however, no previous study has compared the postoperative analgesic effect of SFP plasters with oral analgesics. We hypothesized that an SFP plaster would be effective for post-THA pain with minimal adverse effects and studied the analgesic and adverse effects of applying an SFP plaster after THA.

## Materials and methods

This study was approved by the Ethics Committee of our institution. The need for informed consent was waived by the ethics committee due to the retrospective nature of the study. Of 112 patients who underwent primary THA by the same surgeon at our department between November 2015 and December 2017, 100 patients who met the following criteria were included. The study population consisted of 86 women and 14 men, of which 89 had experienced osteoarthritis of the hip and 11 showed idiopathic femoral head necrosis. All patients underwent muscle-sparing joint replacement with the anterolateral approach using cementless implants on both the acetabular and femoral sides (age at surgery, 62.3 ± 8.9 years (mean ± standard deviation)). Patients who underwent THA with other approaches, such as lateral or posterolateral approaches with muscle-tendon dissection, were excluded. We also excluded patients who had undergone non-elective surgery and those who received urgent or semi-urgent THA for trauma, such as femoral neck fracture. Moreover, patients with preoperative gastrointestinal disturbance, renal dysfunction, or cardiovascular disorders were excluded.

Post-THA analgesia in our department consisted of intraoperative local infiltration of anesthesia with 100 mg of levobupivacaine after implant replacement and intravenous patient-controlled analgesia at 1 mL/hour of a mixture of fentanyl 10 mL, droperidol 1 mL, and physiological saline 39 mL for two days after surgery. Group A consisted of 50 patients who underwent treatment between November 2015 and November 2016 and received oral administration of the selective COX-2 inhibitor celecoxib 400 mg/day (200 mg each after breakfast and after dinner) and the potassium-competitive acid blocker vonoprazan 10 g/day for 14 days from the day after surgery. Group B consisted of 50 patients who underwent treatment between December 2016 and December 2017 and received the SFP plaster (LOQOAⓇ tape 40 mg; 40 mg/140 cm^2^) once daily (after breakfast) for 14 days from the day after surgery. The patients were instructed to apply one plaster at the most painful site, which was usually the anterior surface of the proximal thigh, although some applied it to the buttocks.

We investigated the changes in pain using the numerical rating pain intensity scale (NRS) score, changes in body temperature, and any adverse effects of analgesics. The pain was graded by the NRS score on an 11-point scale from 0 to 10, with 0 representing no pain and 10 the maximum pain imaginable. Patients were asked to grade their pain verbally three times a day (morning, noon, and night) from postoperative day one to day 14, and the highest value for each day was used. Their temperature was also taken three times a day preoperatively and from postoperative days 1-14. The highest values of body temperature were used. Serum creatinine concentration was measured preoperatively and on postoperative days one, three, seven, and 14. Renal function was evaluated by measuring serum creatinine concentration preoperatively and on postoperative days one, three, seven, and 14 and by calculating each patient’s estimated glomerular filtration rate (eGFR) using their sex, age, and serum creatinine level; postoperative renal dysfunction was defined by an eGFR ≤80% of the preoperative value during the postoperative course. Adverse effects at the application site, gastrointestinal disturbances, cardiovascular disorders, and other systemic side effects were diagnosed on the basis of medical histories and physical examinations. Oral acetaminophen was administered as a rescue antipyretic analgesic, and the number of patients who required the rescue medication was counted.

Differences in the mean age, height, weight, preoperative eGFR, and surgery time between Groups A and B were analyzed using the Mann-Whitney U test. The repeated-measure endpoints (NRS score and body temperature) were analyzed with linear mixed models that included the group (A or B), dummy variables for time, group-by-time interactions, and the baseline value of each endpoint as covariates (NRS score on day one, body temperature preoperatively) and the patients as a random effect. The covariance structure was a completely general covariance matrix. The results were reported as the least-squares means with a 95% confidence interval at each time point. A p-value of <0.05 was considered statistically significant, and all p-values were two-sided without multiplicity adjustment. All statistical analyses were performed using SPSS Statistics for Windows/Macintosh (IBM Corp., IBM SPSS Statistics for Windows, version 23.0, Armonk, NY).

## Results

**Figure 1:**
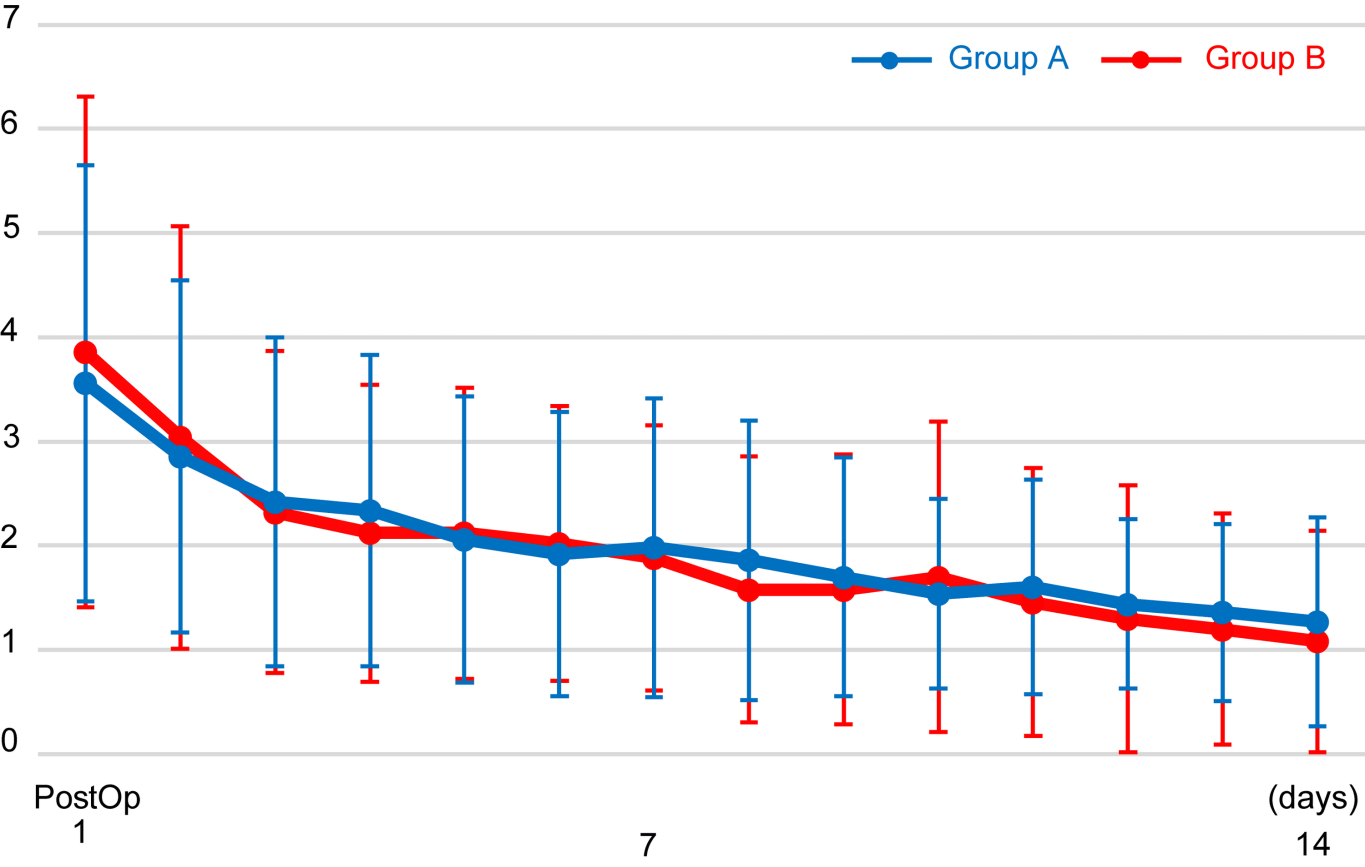
NRS scores from postoperative days 1-14 No significant difference was observed between Groups A and B. NRS: numerical rating pain intensity scale

**Figure 2:**
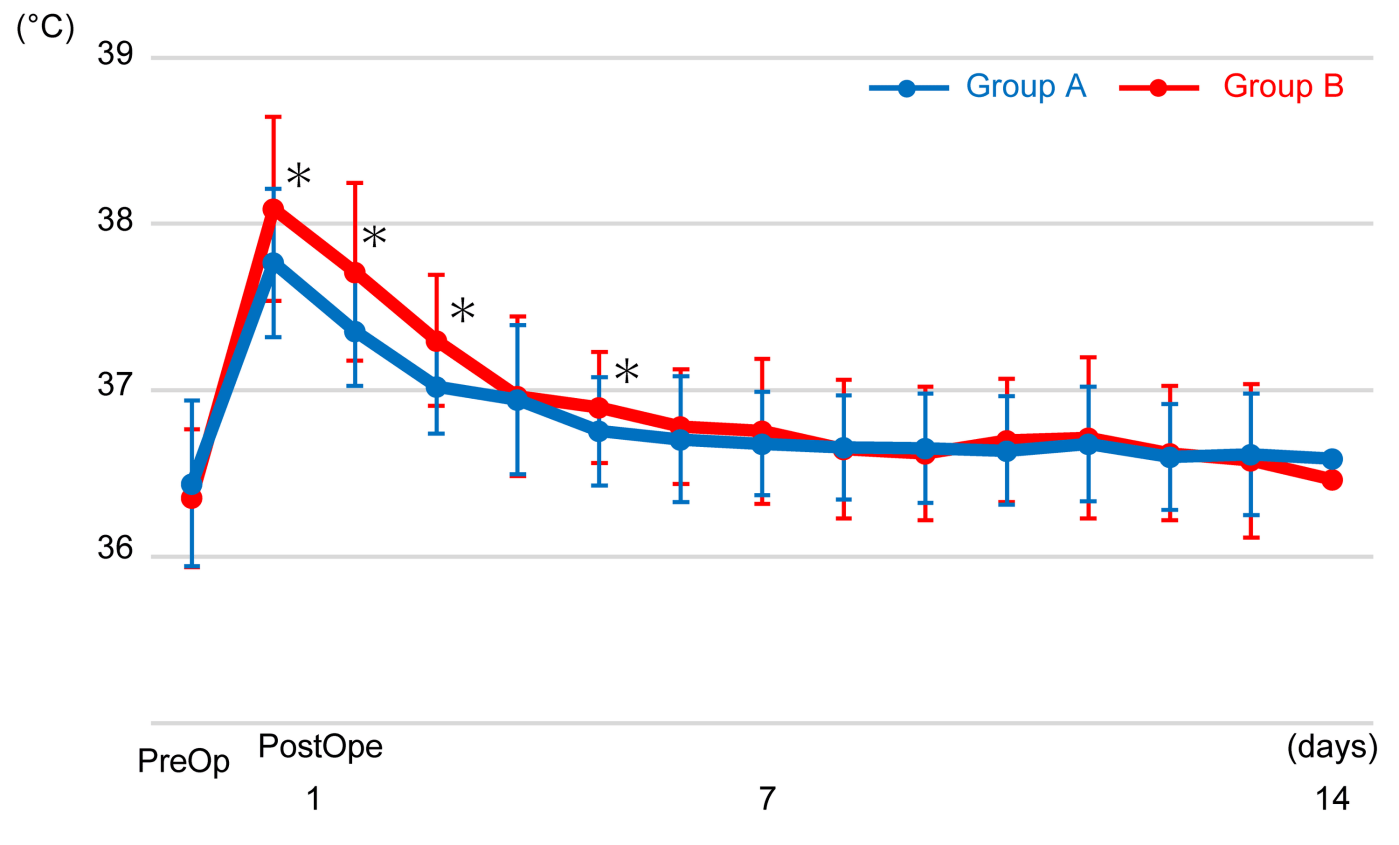
Body temperature obtained preoperatively and from postoperative days 1-14 Body temperature was significantly higher in Group B on postoperative days one, two, three, and five (*p < 0.01).

There were no significant differences in the mean age, height, weight, preoperative eGFR, and surgery time between Groups A and B (p > 0.05) (Table 1). Groups A and B showed no significant differences in the transition of NRS scores (p > 0.05) (Figure 1). The mean NRS score dropped to below 3 points on day two in Group A and on day three in Group B and to below 2 points on day six in Group A and day seven in Group B. The transition of body temperature showed a significant intergroup effect (p < 0.001). Body temperature was significantly higher in Group B than in Group A on days one, two, three, and five (p < 0.01) (Figure 2).

**Table 1:** Patient demographics Data are presented as mean ± standard deviation. eGFR, estimated glomerular filtration rate

	Group A	Group B
Sex		
Male	7 (14%)	7 (14%)
Female	43 (86%)	43 (86%)
Disease		
Osteoarthritis	44 (88%)	45 (90%)
Femoral head necrosis	6 (12%)	5 (10%)
Age (years)	61.3 ± 9.9	62.6 ± 7.8
Height (cm)	156.6 ± 6.4	156.6 ± 6.9
Weight (kg)	59.0 ± 9.3	59.1 ± 11.2
Preoperative eGFR (mL/min)	87.6 ± 22.9	86.1 ± 17.9
Surgery time (min)	114.6 ± 21.0	108.3 ± 26.2

In Group A, two patients (4%) experienced renal dysfunction, and one patient (2%) experienced gastrointestinal disturbance necessitating withdrawal of the medication. However, patients in Group B did not experience any systemic or local adverse effects. Neither group did not show any cardiovascular or other systemic adverse effects.

In Group B, 18 patients (36%) required 400 mg of rescue acetaminophen for fever (17 cases) or headache (one case) management. Acetaminophen for fever was administered only a few times, at most once a day from days one to three, and no patient required its regular use. No patient in Group A required rescue acetaminophen (Table 2).

**Table 2:** Systemic and local adverse effects in both groups In Group A, two patients experienced renal dysfunction, and one patient experienced gastrointestinal disturbance. However, patients in Group B did not experience any systemic or local adverse effects.

Adverse effects	Group A	Group B
Application site	0	0
Renal dysfunction	2 (4%)	0
Gastrointestinal disturbance	1 (2%)	0
Cardiovascular disorders	0	0
Rescue acetaminophen	0	18 (36%)

## Discussion

In this study, we compared the analgesic effect of SFP plaster 40 mg after THA with that of a dose of 400 mg/day of oral celecoxib and found that both were almost equivalent. On a visual analogue pain intensity scale, a score of 30 mm or more is considered to represent moderate pain [18], and the ideal goal is to control pain to below a score of 20 mm. In both groups in the present study, the scores declined to below 3 points within three days and below 2 points within a week, indicating good pain control in both groups. Although several researchers have reported their experience of using an SFP plaster on the knee [15,16,19], there are no studies describing its use on the hip. Our study suggests that the SFP plaster may provide adequate tissue penetration and analgesia even in areas with comparatively thicker subcutaneous fat, such as the hip.

The adverse effects of SFP plaster include its local effects at the site of application, such as dermatitis, erythema, and rash, and systemic adverse effects attributable to its characteristic effective penetration into plasma. No local adverse effects of SFP plaster were observed in this study. In a study of local adverse effects in patients who applied SFP plaster continuously for 52 weeks, 47% showed skin symptoms [20], but the rate was not significantly different from that for the placebo [19]. Plaster application is generally considered to cause severe physical irritation when it is pulled off [21], and rather than the active ingredient SFP, skin symptoms are believed to be due to the physical irritation caused by repeated application and removal; thus, patients must be instructed to pull the tape off gently. Since the present study only covered two weeks, no patient developed local adverse effects. Attention must be paid to skin symptoms when the tape is used for longer periods.

The most important systemic side effects of SFP plaster application are the same as those of oral NSAIDs, including gastrointestinal disturbances, renal dysfunction, and cardiovascular disorders [22]. The reported risk of upper gastrointestinal bleeding due to oral nonselective NSAIDs is four- to fivefold greater than that in patients not taking them [6,23]. Although the selective COX-2 inhibitor celecoxib causes fewer gastrointestinal problems than nonselective NSAIDs [23,24], administration of gastrointestinal protective agents is recommended in patients with high gastrointestinal risk [25]. In this study, despite the use of the potassium-competitive acid blocker vonoprazan to prevent gastrointestinal disturbance during celecoxib use, one patient (2%) experienced gastrointestinal disturbance sufficiently severe to require celecoxib withdrawal. Meanwhile, there were no cases of gastrointestinal disturbance among the patients who used SFP plaster. The reported incidence of gastrointestinal adverse effects associated with long-term use of oral FP is 9.1% [26], in comparison with the 3% and 6% reported for the long-term use of 40 mg and 80 mg, respectively, of SFP plaster [19]. Since the present study only covered two weeks, no patient developed gastrointestinal disturbance. However, the occurrence of a serious bleeding gastric ulcer requiring laparoscopic hemostasis during the use of SFP plaster has been reported [19], and therefore, caution is required.

On the other hand, selective COX-2 inhibitors can cause renal dysfunction [27] and cardiovascular side effects [28] with prolonged use. Because COX-2 is permanently expressed in the kidneys, the incidence of renal dysfunction caused by selective COX-2 inhibitors and nonselective NSAIDs is not different, and a similar level of caution with respect to this adverse effect is required for both categories of drugs [29]. In this study, the eGFR decreased to ≤ 80% of its preoperative value in two patients (4%) taking celecoxib. Because prophylactic antibacterial agents were also administered postoperatively, we could not determine which agent was the primary cause of renal dysfunction. However, this is an important side effect of NSAIDs and must always be considered during their use. A significant elevation in creatinine concentration in week 44 during a 52-week continuous use of SFP plaster has been reported, but the elevated concentration was still within normal limits and did not pose a clinical threat [30]. In addition, no case of serious renal dysfunction was reported.

In this study, we also investigated the body temperature, and the mean temperature from postoperative days one to five was higher in patients who used the SFP plaster than in those who took celecoxib, with 17 patients (34%) in Group B requiring rescue acetaminophen. This suggests that the SFP plaster has minimal systemic antipyretic effects. Although the systemic exposure during the use of SFP plaster 80 mg is roughly equivalent to that associated with the normal dose of oral FP, the maximum plasma concentration is only 66% [22,26], and the gradual and continuous absorption may help mitigate the systemic adverse effects. The problem of postoperative fever can be solved by taking oral acetaminophen regularly for several days after the surgery.

The most critical limitation of this study is the retrospective design. However, there were no significant differences in the mean age, height, weight, preoperative eGFR, and surgery time between the two groups. Moreover, both groups were treated by the same process, except for postoperative analgesic drugs. Although rescue acetaminophen was used for fever or headache in Group B, we considered that it did not affect the highest value of NRS scores because it was used for once a day at most. Another limitation is that only THA by the anterolateral approach was included in this study. The comparison between SFP plaster and celecoxib may have shown significant differences in NRS scores in cases involving more invasive THA, including the muscle-tendon dissection approach or revision surgery. However, the anterolateral approach is the most commonly performed procedure in our department, and the postoperative analgesic effect achieved using SFP plaster after this procedure was almost the same as that achieved with oral administration of celecoxib. Therefore, we consider that SFP plaster is an effective dosage form for postoperative analgesia.

## Conclusions

To our knowledge, this is the first study to compare the postoperative analgesic effect of SFP plasters with oral analgesics. In post-THA patients, its analgesic effect was similar to that of the widely used celecoxib. Additionally, there were no obvious side effects. Therefore, we consider that the SFP plaster application is an effective approach for postoperative analgesia.
